# Longitudinal changes in clinical characteristics and outcomes for children using long-term non-invasive ventilation

**DOI:** 10.1371/journal.pone.0192111

**Published:** 2018-01-30

**Authors:** Maria L. Castro-Codesal, Kristie Dehaan, Prabhjot K. Bedi, Glenda N. Bendiak, Leah Schmalz, Sherri L. Katz, Joanna E. MacLean

**Affiliations:** 1 Department of Pediatrics, University of Alberta, Edmonton, Alberta, Canada; 2 Women & Children’s Health Research Institute, University of Alberta, Edmonton, Alberta, Canada; 3 Stollery Children’s Hospital, Edmonton, Alberta, Canada; 4 Department of Pediatrics, University of Calgary, Calgary, Alberta, Canada; 5 Alberta Children’s Hospital, Calgary, Alberta, Canada; 6 Department of Pediatrics, University of Ottawa, Ottawa, Ontario, Canada; 7 Children’s Hospital of Eastern Ontario, Ottawa, Ontario, Canada; Boston Children's Hospital / Harvard Medical School, UNITED STATES

## Abstract

**Objectives:**

To describe longitudinal trends in long-term non-invasive ventilation (NIV) use in children including changes in clinical characteristics, NIV technology, and outcomes.

**Methods:**

This was a multicenter retrospective cohort of all children started on long-term NIV from 2005 to 2014. All children 0 to 18 years who used NIV continuously for at least 3 months were included. Measures and main outcomes were: 1) Number of children starting NIV; 2) primary medical condition; 3) medical complexity defined by number of comorbidities, surgeries and additional technologies; 4) severity of sleep disordered breathing measured by diagnostic polysomnography; 5) NIV technology and use; 6) reasons for NIV discontinuation including mortality. Data were divided into equal time periods for analysis.

**Results:**

A total of 622 children were included in the study. Median age at NIV initiation was 7.8 years (range 0–18 years). NIV incidence and prevalence increased five and three-fold over the 10-year period. More children with neurological and cardio-respiratory conditions started NIV over time, from 13% (95%CI, 8%-20%) and 6% (95%CI, 3%-10%) respectively in 2005–2008 to 23% (95%CI, 18%-28%) and 9% (95%CI, 6%-14%, p = 0.008) in 2011–2014. Medical complexity and severity of the sleep-disordered breathing did not change over time. Overall, survival was 95%; mortality rates, however, rose from 3.4 cases (95% CI, 0.5–24.3) to 142.1 (95% CI 80.7–250.3, p<0.001) per 1000 children-years between 2005–2008 and 2011–2014. Mortality rates differed by diagnostic category, with higher rates in children with neurological and cardio-respiratory conditions.

**Conclusions:**

As demonstrated in other centers, there was a significant increase in NIV prevalence and incidence rate. There was no increase in medical complexity or severity of the breathing abnormalities of children receiving long-term NIV over time. The mortality rate increased over time, maybe attributable to increased use of NIV for children with neurological and cardio-respiratory conditions.

## Introduction

Non-invasive ventilation (NIV) is a method of ventilatory support that has increasingly been used for a range of respiratory and sleep disorders in children since the first reports in the 1980’s [1–3]. With technological advances in NIV, where positive pressure is delivered through an interface outside the airway, children and their families sometimes have an alternative to invasive ventilation, where positive pressure is delivered through an endotracheal or tracheostomy tube [4]. Over the last two decades, the use of NIV has led to a 5-15-fold increase in the use of home mechanical ventilation worldwide, with worldwide prevalence ranging from 2.1 to 13.7/100,000 in children [5–13]. Today, NIV is considered the standard of care for a range of medical conditions leading to sleep disordered breathing and chronic respiratory insufficiency or failure with a greater proportion of those starting on home mechanical ventilation surviving to adulthood [6, 14].

While the efficacy of long-term NIV for many underlying conditions is well established, [14] information documenting the expanded use of this technology worldwide is limited to cross-sectional studies or single-centered cohorts [5–10, 12, 13, 15–18]. Moreover, most of the aforementioned studies describe the increase in use with very little information about trends of clinical characteristics, NIV technology and use, or discontinuation rates. Multi-centered studies measuring longitudinal outcomes of long-term NIV programs are lacking. The objectives of this longitudinal multi-centered study are to: (1) describe the longitudinal trends of pediatric long-term NIV use over a 10-year period; and (2) examine the changes in clinical characteristics, NIV technology use, and long-term outcomes including NIV discontinuation and mortality rates. We hypothesized that, as NIV use has become more common, there has been a greater diversity, higher complexity, and increased severity of sleep disordered breathing in children receiving NIV. Understanding the changes in NIV use over time will help us determine the best use of this technology and plan for future health care needs.

## Materials and methods

### Study design

This multicenter, regional, longitudinal population study examined children using long-term NIV in the province of Alberta and surrounding provinces between January 2005 and December 2014. The two tertiary care children’s hospitals in the province, the Stollery Children’s Hospital (Edmonton) and the Alberta Children’s hospital (Calgary), participated in the study. As these hospitals also house the only government funded pediatric sleep laboratories, our cohort represents the majority of, if not all, children using NIV in the province of Alberta. These programs also provide care for children in surrounding provinces, including the Northwest Territories, and parts of Saskatchewan, British Columbia, and the Yukon.

The study protocol was approved by the Health Research Ethics Board (HREB) at the University of Alberta and the Conjoint Health Research Ethics Board (CHREB) at the University of Calgary. As this was a retrospective study, our research ethics board waived the need for consent from participants and legal guardians. Subject identifiers were collected to enable data matching, but subjects were assigned a subject number during the data extraction and identifiers were stored separately from the research data. Data was stored in a secure REDCap electronic database [19].

### Measures of interest

Changes in the following measures were assessed: 1) Incidence and prevalence of children on long-term NIV; 2) Primary medical conditions; 3) Medical complexity of children defined by the total number of comorbidities, surgeries, and use of additional technologies; 4) Severity of sleep disordered breathing measured by diagnostic polysomnography (PSG); 5) NIV technology use; and 6) Reasons for NIV discontinuation including mortality.

### Subjects

Subjects included all children aged 0 to 18 years receiving NIV in a non-acute care setting for at least 3 months continuously. We defined NIV as any mode of ventilatory support where positive pressure is delivered through an interface outside the airway, including continuous positive airway pressure (CPAP), auto positive airway pressure (auto-PAP) and bi-level positive airway pressure (BPAP) therapies. Subjects were identified through the hospital records of the NIV programs at the two participating hospitals for all children referred for NIV initiation.

### Data collection

Data collection included review of medical charts and sleep laboratory records. Data on ethnicity were self-reported by patients/parents at one centre, and physician reported at the other. Demographics, primary diagnoses, chronic comorbidities, any surgery performed prior to NIV initiation that was documented in the medical chart (tympanostomy tubes and dental restoration were excluded from the analysis), and other additional technologies in use were collected at the time of NIV initiation. The primary conditions leading to the need for NIV initiation were grouped into five broad diagnostic categories: central nervous system (CNS), upper airway (UA), cardio-respiratory (Cardio-Resp; excludes UA), musculoskeletal and neuromuscular disorders (MSNM), and unclassified conditions. Children with multiple medical conditions were grouped as ‘unclassified’ if it was not possible to identify the specific medical condition leading to NIV initiation. Any other chronic co-occurring medical diagnoses documented in the chart expected to be long-term or potentially lifelong were considered comorbidities [20]. Data extracted from the diagnostic PSG or the diagnostic portion of a split PSG, including the number of apneas and hypopneas per hour of sleep, oxygen saturation and carbon dioxide levels, were used to assess the severity of the sleep disordered breathing. Data on NIV initiation included NIV technology and settings, interface type, triggers for starting NIV, location for NIV initiation, and number of used hours. Compliance data, including the number of nights with use for more than 4 hours and average number of hours per night, was extracted from NIV machine downloads. Reasons for NIV discontinuation and total duration of NIV in those children discharged from the NIV programs were collected at the most recent visit.

### Statistical analysis

Descriptive statistics summarized patient characteristics, NIV technology and reasons for NIV discontinuation. Incidence rate (per 100,000 children per year) and prevalence (per 100,000 children) for children living in Alberta over a 10-year period were calculated using data available from Census Canada on the number of children 0–19 years of age [21]. Changes in NIV annual incidence rate were calculated using permutation tests for joinpoint regression model. [22] For the rest of the trend analysis, data were divided into three equal 3.3-year epochs (2005–2008; 2008–2011; 2011–2014) to calculate differences over time. Kruskal-Wallis test was used to compare differences in medians of non-normally distributed variables over time. Post hoc Bonferroni correction was applied for multiple comparisons. Pearson Chi-Square and Fisher’s Exact Test were used to assess differences between categorical variables. Trend analysis was used to calculate trends in outcome rates over time. Survival curves and log-rank test were used to estimate differences in survival by diagnostic category. Hypothesis tests were 2-sided and statistically significant differences between groups were documented by p < 0.05. SPSS version 24.0 (1989, 2016), STATA v13 (STATA, 2013) and the Joinpoint Regression Program (REF) were used for statistical analysis.

## Results

### Description of patient characteristics

A total of 891 records were reviewed; 216 children used NIV for less than three months or were older than 18 at NIV start, and 51 charts were unavailable. The analysis included 622 children (61% male) of whom 87% were living in Alberta (Table 1). The median age at NIV initiation was 7.8 y (0–18 y) with 18% of children <2 years, 16% from 2 to 4.9 years, 29% from 5 to 11.9 years and 29% over 12 years. The most common ethnicity (data available for 387 children) was Caucasian (268, 69%) followed by Aboriginal (45, 12%), Asian (40, 10%), African (17, 4%), Latin American (6, 2%), and mixed ethnicity (11, 3%). UA was the most common diagnostic category (60%) followed by CNS (17%). Details of the underlying conditions are available in Table 2. One or more comorbidities were identified in 92% of children. After adeno ± tonsillectomy, gastrostomy tube and/or fundoplication were the most common surgery prior to starting NIV. One or more additional technologies were used by 25% of children.

**Table 1:** Clinical characteristics of 624 children started on long-term non-invasive ventilation.

Patient characteristics	n = 622 n (%); median (range)
**Diagnostic category**	
UA	371 (60)
CNS	107 (17)
MSNM	93 (15)
Cardio-Resp	39 (6)
Unclassified	12 (2)
**Number of comorbidities**	
0	50 (8)
1–2	310 (50)
3–4	161 (26)
5 or more	101 (16)
**Surgeries prior to starting NIV**	
AT/ adenoidectomy / tonsillectomy	300 (48)
G-tube and/or fundoplication	90 (15)
Neurosurgery	52 (8)
Cardiac	50 (8)
Upper airway	41 (7)
Spinal	21 (3)
Tracheostomy	19 (3)
Orthognatic surgery	8 (1)
**Additional technologies**	
G-tube/ NG tube feeding	99 (16)
Wheelchair	63 (10)
Daytime oxygen	30 (5)
V-P shunt	17 (3)
**Pre-NIV diagnostic PSG** ^a^	
AHI, events/hour	11.2 (0–238)
Mean SpO_2_, %	94.8 (64–99.9)
Mean ETCO_2_, mmHg	44.7 (30.4–72.4)
Mean TcCO_2_, mmHg	44.6 (32–99.3)

AHI, Apnea-Hypoapnea index; AT, adenotonsillectomy; Cardio-Resp, cardio-respiratory (excludes UA); CNS, central nervous system; ETCO_2_, end-tidal carbon dioxide; G-tube, gastrostomy tube; MSNM, musculoskeletal and neuromuscular; NG, nasogastric; PSG, polysomnography; SpO_2_, pulse oxygen saturation; TcCO_2_, transcutaneous carbon dioxide; UA, upper airway; V-P, shunt, ventriculo-peritoneal shunt.

^a^ Data available for 547 (87%) children.

**Table 2:** Diagnostic categories and disease subgroups leading to initiation of non-invasive ventilation. (adapted from Wallis, 2011) [13].

Diagnostic categories (%)	Disease subgroup	n = 622
Central Nervous System (17%)	Congenital brain lesion	50
	Acquired brain injury	12
	Brain tumor	11
	Metabolic disease	8
	Congenital central hypoventilation syndrome	6
	Other central causes	20
Musculoskeletal and neuromuscular (15%)	Congenital myopathies	33
	Achondroplasia	17
	Duchenne muscular dystrophy	16
	Spinal muscular atrophy type 1, 2, 3	11
	Other muscular dystrophies	6
	Myelomeningocele	5
	Mucopolysaccharidosis	5
Upper airway (60%)	Obesity	119
	Down syndrome	111
	Obstructive sleep apnea	56
	Upper airway narrowing/malformation	35
	Airway malacia	32
	Craniosynostosis	7
	Prader Willi	11
Cardio- respiratory (6%)	BPD	13
	Chronic lung disease	10
	CHD	6
	Pulmonary hypertension	4
	Cystic fibrosis	3
	Cardiac failure	3
Unclassified (2%)	Chromosomal abnormality Diaphragmatic hernia and brain injury Fanconi syndrome Albright hereditary osteodystrophy BPD, CHD and myopathy Ectodermal dysplasia Diagnosis not available	4 1 1 2 1 1 2

Children with multiple medical conditions were allocated to a specific diagnostic category according to the medical condition that required non-invasive ventilation. If it was not possible to identify the specific medical condition leading to NIV initiation, children were grouped as ‘unclassified’. BPD, bronchopulmonary dysplasia; CHD, Congenital heart disease.

### Description of NIV technology, use and discontinuation

CPAP was the most common NIV type (75%) followed by BPAP (22%) and auto-PAP (1%). Of the 550 children (88%) where data on mask interface was available, 62% of them used a nasal interface, 37% a full face mask, and 1% other interfaces (i.e. total face mask, nasal pillows). NIV was started electively (before or after a PSG) for 83% of children, while 16% started during an acute illness, and 1% due to forced vital capacity below 30%, change from invasive ventilation to NIV, or as part of palliative care treatment. The majority of children started NIV at home (82%) with 18% starting in hospital. NIV was used primarily during nocturnal sleep (86%) followed by during nocturnal sleep and naps (9%), and for sleep and wake times (5%). Median airway pressure for CPAP was 7 (range 4–20) mmHg and median inspiratory and expiratory pressures for BPAP were 15 (range 8–22) mmHg and 6 (range 4–15) mmHg respectively with back-up respiratory rate of 16 (range 0–30) breaths/min. By the end of the study period in December 2014 (760 person-years; median follow-up period was 27 months, range 3–118), 46% of children had continued NIV, 39% discontinued NIV, 14% were transferred to adult services or other respiratory clinics, and 1% was lost to follow-up. For the children that discontinued NIV, the median duration of NIV prior to discontinuation was 21 (range 3–105) months. Reasons for NIV discontinuation were improvement in the underlying condition (16%), patient or family decision to stop therapy (15%), death (5%), switch to IMV (1%), or other reasons (3%). Other reasons included physician recommendation due to interaction with other therapies (i.e. oral appliance or maxillary-facial surgery) or physician recommendation due to poor quality of life. The median age at death was 3.4 y (range 0.25–20.9 y); the primary medical conditions of children who died are presented in Table 3.

**Table 3:** Summary of deaths in children using non-invasive ventilation by diagnostic categories.

Diagnostic category n deaths/total n (%)	Specific disease	n = 28
Central Nervous System n = 14/107 (13%)	Congenital brain lesion	8
	Metabolic disease	3
	Brain tumor	1
	Congenital central hypoventilation	1
	Lennox-Gateaux syndrome	1
Musculoskeletal and neuromuscular n = 6/93 (6%)	Congenital myopathy	2
	Duchenne muscular dystrophy	2
	Spinal muscular atrophy type 1	1
	Spinal muscular atrophy type 2	1
Upper airway n = 3/371 (1%)	Down syndrome	1
	Obstructive sleep apnea	1
	Pfeiffer syndrome	1
Cardio-respiratory n = 5/39 (13%)	Congenital heart disease	3
	Cystic fibrosis	1
	Cardiac failure	1

### Longitudinal trends in incidence and prevalence of NIV

**Fig 1:**
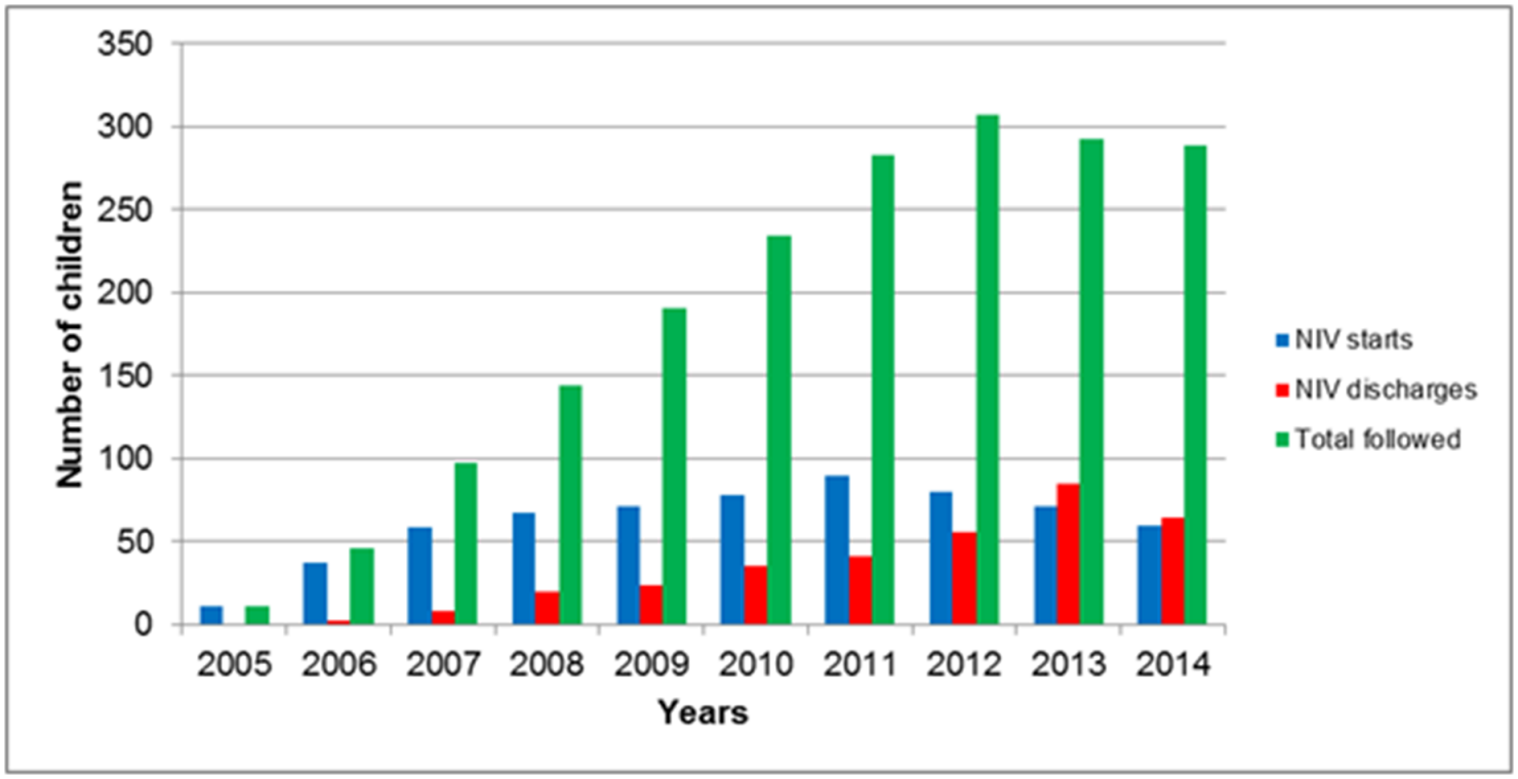
New NIV starts, discharges and total number of children followed by the NIV programs. NIV, non-invasive ventilation.

Annual NIV incidence rate (excluding children from other provinces) increased significantly each year during the period 2005–2008, from 1.65 per 100,000 children started on NIV in 2005 to 8.01 in 2008 and then stabilized at 7.9 per 100,000 children started on NIV per year during the periods 2008–2011 and 2011–2014 (Regression joint point model, p<0.001; Fig 1). The number of children transferred to adult services increased over time, with a median of zero (range 0–1) children/y between 2005–2008, two (range 0–9) children/y between 2008–2011, and four (range 5–19) children/y between 2011–2014 (Kruskal-Wallis test, p<0.001). Post hoc analysis revealed differences between 2005–2008 and 2011–2014 (adjusted p = 0.01). Because of changes in incidence and discharge rates, the prevalence rose from 10.3 children on NIV (95% CI, 10.2 to 10.3) per 100,000 children in the period 2005–2008 to 27.2 (95% CI, 27.1 to 27.3) in the period 2008–2011, to 27.9 (95% CI, 27.8 to28) in the period 2011–2014 (Kruskal-Wallis test, p = 0.007), with post hoc analysis demonstrating an increase in prevalence between the periods 2005–2008 and 2011–2014 (adjusted p = 0.005).

### Longitudinal trends in patient characteristics

The proportion of children in each disease categories changed across epochs, with a higher proportion of children with CNS and Cardio-Resp conditions in the period 2011–2014, and a drop in the proportion of children with MSNM conditions (Table 4). The proportion of children who had adenoidectomy and/or tonsillectomy prior to starting NIV decreased over time. Age, number of comorbidities, other surgeries, additional technologies, and respiratory parameters on the initial diagnostic PSG did not change by epoch. Post doc subgroup analysis by diagnostic category showed no changes in the number of comorbidities, surgeries, additional technologies and respiratory parameters of the PSG over time, except for mean SpO2 in children with CNS conditions, with values of 94.5, 96 and 94.6% in each epoch respectively (Kruskal-Wallis, p = 0.026).

**Table 4:** Longitudinal trends in the clinical characteristics of children using long-term non-invasive ventilation.

Clinical characteristics	Epoch 1 Jan 2005-Apr 2008 (n = 127)	Epoch 2 May 2008-Aug 2011 (n = 262)	Epoch 3 Sept 2011-Dec 2014 (n = 233)	*P* value
**Age** ^a^, median (range), years	7.5 (0.2–17.9)	8.2 (0–18)	7.8 (0–17.8)	0.90
**Diagnostic category** ^b^; n, %				
UA	80, 63 (95%CI 54–71)	161, 61 (95%CI 55–67)	130, 56 (95%CI 50–62)	0.34
CNS ^c^	17, 13 (95%CI 8.5–20)	37, 14 (95%CI 10–18)	53, 23 (95%CI 18–28)	**0.02**
MSNM ^d^	21, 17 (95%CI 11–24)	49, 19 (95%CI 14–24)	23, 10 (95%CI 7–14)	**0.01**
Cardio-Resp ^e^	7, 6 (95%CI 3–10)	10, 4 (95%CI 2–7)	22, 9 (95%CI 6–14)	**0.03**
Unclassified	2, 2 (95%CI 0.4–5)	5, 2 (95%CI 0.8–4)	5, 2 (95%CI 0.9–5)	1
**Comorbidities** ^b^; n, %				
0	12, 9 (95%CI 6–16)	18, 7 (95%CI 4–11)	20, 9 (95%CI 6–13)	0.64
1–2	57, 45 (95%CI 37–54)	138, 53 (95%CI 47–59)	115, 49 (95%CI 42–55)	0.34
3–4	39, 31 (95%CI 23–39)	66, 25 (95%CI 20–31)	56, 24 (95%CI 19–30)	0.37
5 or more	19, 15 (95%CI 10–22)	40, 15 (95%CI 11–20)	42, 18 (95%CI 13–24)	0.59
**Surgeries** ^b^; n, %				
AT/Adenoidectomy ^f^	74, 58 (95%CI 50–67)	133, 51 (95%CI 45–57)	88, 37 (95%CI 32–44)	<**0.001**
Other surgeries	50, 39 (95%CI 31–48)	96, 37 (95%CI 31–43)	85, 37 (95%CI 31–43)	0.86
**Additional technologies** ^b^; n, %				
Daytime oxygen	8, 6 (95%CI 3–12)	12, 5 (95%CI 3–8)	10, 4 (95%CI 2–8)	0.71
Wheelchair	16, 13 (95%CI 8–20)	22, 8 (95%CI 6–12)	25, 11 (95%CI 7–15)	0.40
G/NG tube feeding	19, 15 (95%CI 10–22)	40, 15 (95%CI 11–20)	40, 17 (95%CI 13–23)	0.75
V-P Shunt	3, 2 (95%CI 0.8–7)	7, 3 (95%CI 1–5)	7, 3 (95%CI 1–6)	1
**Diagnostic PSG** ^a^, median (range)				
AHI, events/hour	10 (0–197.2)	12.3 (0.4–200)	11 (0–237.9)	0.45
Mean SpO_2_, %	94.7 (64–98)	95.1 (74.8–99)	94.6 (73.8–99.9)	0.34
Mean ET CO_2_, mmHg	45.1 (30.4–55.8)	44.7 (31.6–72.4)	44.2 (33.9–61.5)	0.96
Mean Tc CO_2_, mmHg	45.6 (34.3–65)	44.9 (35.3–99.3)	44.2 (32–72.4)	0.19

AHI, Apnea-Hypoapnea index; AT, adenotonsillectomy; Cardio-Resp, cardio-respiratory (excludes UA); CNS, central nervous system; ETCO_2_, entidal carbon dioxide; G-tube, gastrostomy tube; MSNM, musculoskeletal and neuromuscular; NG, nasogastric; PSG, polysomnography; SpO_2_, pulse oxygen saturation; TcCO_2_, transcutaneous carbon dioxide; UA, upper airway; V-P Shunt, ventriculo-peritoneal shunt.

^a^ Kruskal-Wallis test.

^b^ Pearson Chi-Square test or Fisher’s Exact test.

^c^ Adjusted residuals for CNS were -1.3, -1.7 and 2.8 in each period respectively.

^d^ Adjusted residuals for MSNM were 0.6, 2.3 and -2.8 in each period respectively.

^e^ Adjusted residuals for Cardio-Resp were -0.4, -2.1 and 2.5 in each period respectively.

^f^ Adjusted residuals for adenotonsillectomy were 2.7, 1.5 and -3.8 in each period respectively.

### Longitudinal trends in NIV technology and use

The use of nasal masks increased, with a concomitant decrease in full face masks over time (Table 5). Median CPAP pressure increased while BPAP inspiratory positive airway pressure and respiratory rate decreased over time. NIV use during sleep versus sleep and awake did not change over time. Indication and location for NIV initiation as well as the NIV mode were not different across epochs.

**Table 5:** Longitudinal trends in the technology for children using long-term non-invasive ventilation.

	Jan 2005-Apr 2008 (n = 127)	May 2008-Aug 2011 (n = 262)	Sept 2011-Dec 2014 (n = 233)	*P* Value
**Trigger for NIV** ^a^; n, %				
Electively with PSG	90, 71 (95%CI 62–78)	193, 74 (95%CI 68–79)	170, 73 (95%CI 67–78)	0.85
Electively without PSG	19, 15 (95%CI 10–22)	22, 8 (95%CI 6–12)	18, 8 (95%CI 5–12)	0.05
Acute illness	16, 13 (95%CI 8–19)	43, 17 (95%CI 12–21)	40, 17 (95%CI 13–23)	0.48
Other ^b^	2, 1 (95%CI 0.4–6)	2, <1 (95%CI 0.2–3)	4, 2 (95%CI 0.6–4)	0.62
**Location to start NIV** ^a^; n, %				
Home settings	107, 84 (95% CI 77–90)	214, 82 (95%CI 77–86)	185, 79 (95%CI 74–84)	0.49
PICU	6, 5 (95%CI 2–10)	26, 10 (95%CI 7–14)	26, 12 (95%CI 0.8–16)	0.1
Ward	14, 11 (95%CI 6–18)	21, 8 (95%CI 5–12)	21, 9 (95%CI 6–13)	0.63
**Interface type** ^a^^,^ ^c^; n, %				
Nasal mask ^d^	75, 63 (95%CI 54–72)	109, 47 (95%CI 41–53)	156, 78 (95%CI 73–84)	<**0.001**
Full face mask ^e^	43, 36 (95%CI 28–45)	120, 52 (95%CI 45–58)	40, 20 (95%CI 15–26)	<**0.001**
Other ^f^	1, <1 (95%CI 0.1–5)	5, 2 (95%CI 0.9–5)	1, <1 (95%CI 0.1–3)	0.29
**NIV type** ^a^; n, %				
CPAP	101, 80 (95%CI 72–86)	207, 79 (95%CI 74–84)	171, 73 (95%CI 67–78)	0.19
BPAP	25, 20 (95%CI 14–28)	52, 20 (95%CI 15–25)	62, 26 (95%CI 21–32)	0.17
Auto-PAP	1, <1 (95%CI 0.1–4)	3, 1 (95%CI 0.4–3)	2, <1 (95%CI 0.2–3)	>0.99
**NIV settings** ^g^; Median (range)				
CPAP (cm H_2_O) ^h^	7 (4–13)	8 (4–16)	7 (4–20)	**0.03**
IPAP (cm H_2_O) ^i^	14 (10–22)	14 (9–22)	12 (8–22)	**0.009**
EPAP (cm H_2_O)	5 (4–10)	6 (4–15)	6 (4–12)	0.42
Back-up rate ^j^	18 (0–30)	20 (0–30)	15 (0–30)	**0.002**
**NIV use** ^a^; n, %				
Night sleep	115, 91 (86–96)	227, 87 (82–91)	192, 82 (76–87)	0.09
Night sleep and naps	10, 8 (3–13)	21, 8 (4–12)	24, 10 (5–7)	0.6
Sleep and awake	2, 2 (0–4)	13, 5 (2–8)	17, 7 (2–10)	0.06

Auto-PAP, auto positive airway pressure therapy; BPAP, bilevel positive airway pressure therapy; CPAP, continuous positive airway pressure therapy; EPAP, expiratory positive airway pressure; IPAP, inspiratory positive airway pressure; NIV, non-invasive ventilation; PICU, pediatric intensive care unit; PSG, polysomnography.

^a^ Pearson Chi Square or Fisher’s Exact test.

^b^ Other include failure to wean invasive ventilation, forced vital capacity (FVC) below 30%, and as part of palliative care treatment.

^c^ Data on mask interface available in 119, 232, 199 in each epoch respectively.

^d^ Adjusted residuals for nasal mask were 0.3, -6.4, and 6.3 in each period respectively.

^e^ Adjusted residuals for full face mask were -0.2, 6.1 and -6.1 in each period respectively.

^f^ Other interfaces: total mask, nasal pillows.

^g^ Kruskal-Wallis test.

^h^ Post hoc Bonferoni analysis showed differences between period 2005–2008 and 2008–2011 (adjusted p = 0.03).

^I^ Post hoc Bonferoni analysis showed differences between period 2008–2011 and 2011–2014 (adjusted p = 0.01).

^j^ Post hoc Bonferoni analysis showed differences between period 2008–2011 and 2011–2014 (adjusted p = 0.002).

Compliance data did not change across epochs (Kruskal-Wallis, p = 0.7), with no changes in the percentage of nights with NIV use above 4 hrs (median of 75%, range 0–100%) or in the total number of NIV hours (median 7hrs, range 0.3–20).

### Longitudinal trends of outcomes

The number of children who died while using NIV increased over time, with mortality rates that went from 3.4 cases (95%CI, 0.5 to 24.3) per 1000 children started on NIV-years in 2005–2008 to 39.2 (95%CI, 23.6 to 64.9) in 2008–2011 and 142.1 (95%CI, 80.7 to 250.3) in 2011–2015 (Table 6, adjusted by age at NIV initiation). No changes in age at death occurred over time (Kruskal-Wallis, p = 0.3), with median age of death of 18 years for the single child that died in 2005–2008, 3 years (0.25–20) in 2008–2011 and 4 years (0.8–17.8) in 2011–2014. NIV discontinuation rates due to improvement of the underlying condition, patient/family decision to stop NIV, and transfer to adult services increased over time. There was no change in the rate of children switched to invasive ventilation over time.

**Table 6:** Longitudinal trends in mortality and discontinuation rates for children using long-term non-invasive ventilation.

Reasons for discontinuation	Jan 2005-Apr 2008 (n = 127)	May 2008-Aug 2011 (n = 262)	Sept 2011-Dec 2014 (n = 235)	*P* Value
Mortality	3.4 (0.5–24.3)	39.2 (23.6–64.9)	142.1 (80.7–250.3)	<**0.001**
Family/patient decision to stop NIV	74.1 (48.8–112.5)	118.2 (88.8–157.3)	245.4 (159.9–376.3)	<**0.001**
Improvement	84.2 (56.9–124.6)	123.2 (93.1–163)	292.1 (197.4–432.3)	<**0.001**
Change to invasive ventilation	3.4 (0.5–23)	17.6 (8.4–36.9)	0	0.65
Transfer to adults	74.1 (48.8–112.5)	88 (63.2–122.6)	105.2 (54.7–202.1)	<**0.001**

Rates are expressed as number of cases per 1000 children initiated on NIV in each period per years (95% CI) and adjusted by age. NIV, non-invasive ventilation.

**Fig 2:**
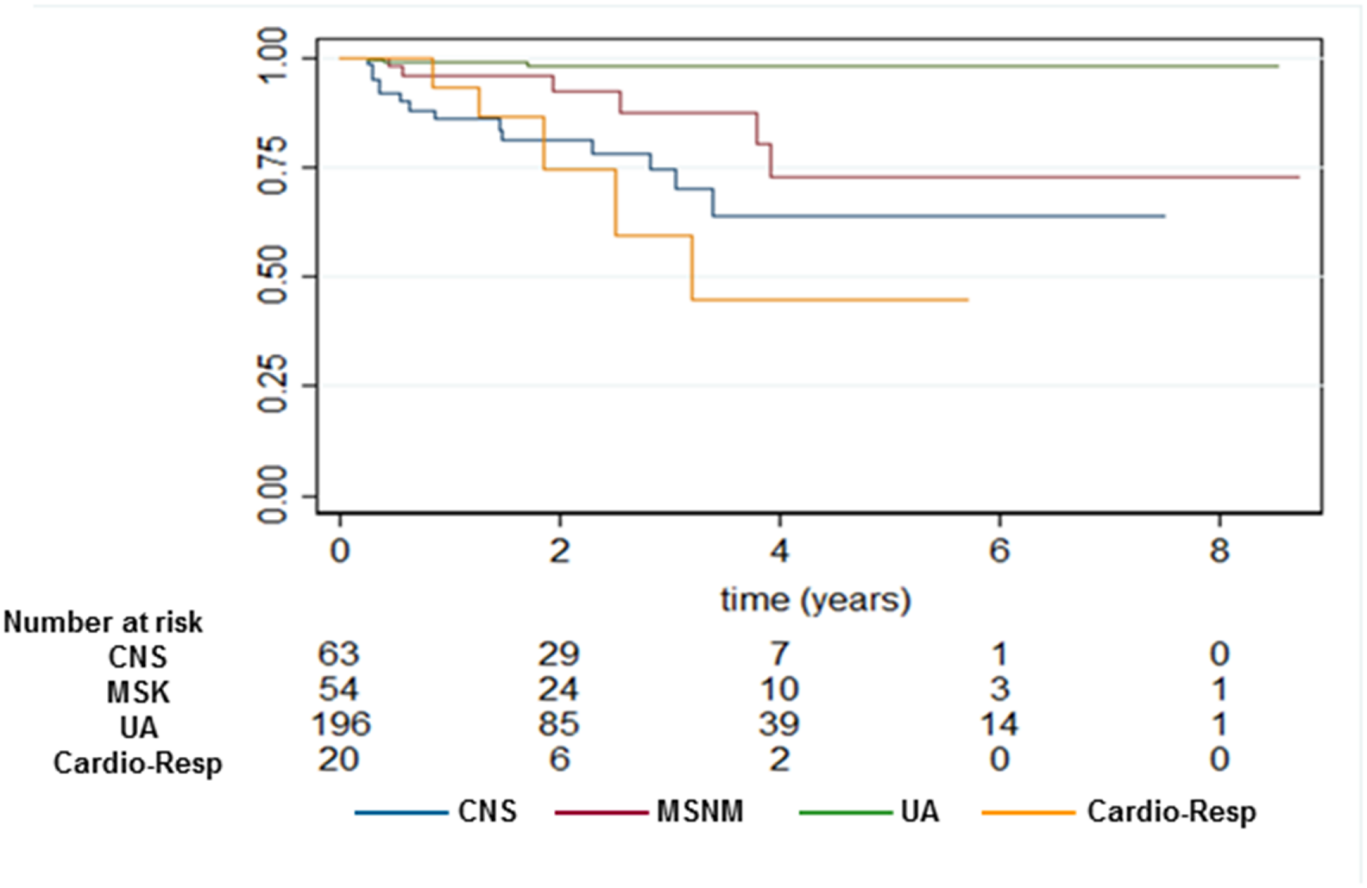
Kaplan-meier survival curves in children on long-tern NIV by diagnostic category. Category “Unclassified” was excluded because there were no deaths in this group. There were significant differences in survival curves by diagnostic category (Log-Rank test, p<0.001). Indicated below, the number of children at risk for death within each diagnostic category per year.

Subgroup analysis showed that survival curves differed by diagnostic category with lower survival in children with Cardio-Resp and CNS conditions, compared to MSNM and UA conditions (Fig 2). Mortality rate increased over time for children with CNS conditions, while mortality rates for other diagnostic categories did not change (Table 7).

**Table 7:** Longitudinal trends in mortality rate for children using long-term non-invasive ventilation within each diagnostic category.

Mortality rate by diagnostic category	Jan 2005-Apr 2008 (n = 127)	May 2008-Aug 2011 (n = 262)	Sept 2011-Dec 2014 (n = 235)	*P* Value
**UA**	0	9.1 (2.3–36.5)	20.9 (2.9–148.1)	0.23
**CNS**	0	87.7 (39.4–195.1)	415 (207.6–829.9)	<**0.001**
**MSNM**	22.5 (3.2–159.9)	52.8 (19.8–140.7)	112.6 (15.9–799.3)	0.13
**Cardio-Resp**	0	152.5 (49.2–472.9)	239.3 (59.9–957)	0.09

Rates are expressed as number of cases per 1000 children initiated on NIV in each period per year (95%CI) and adjusted by age. Cardio-Resp, cardio-respiratory (excludes UA); CNS, central nervous system; MSNM, musculoskeletal and neuromuscular; NIV, non-invasive ventilation; UA, upper airway.

## Discussion

This study describes changes in the incidence, patient characteristics, technology use, and outcomes for a large multicenter cohort of children using long-term NIV. The results demonstrate that the incidence and prevalence of children using long-term NIV grew almost by five and three- fold respectively over the first years and plateaued afterwards. The medical conditions of children using long-term NIV changed with an increase in the total number and proportion of children with CNS and Cardio-Resp conditions and a decrease in the proportion of children with MSNM disorders. Neither medical complexity, defined as the number of chronic comorbidities and additional technology use, nor the severity of sleep disordered breathing by PSG parameters changed over time. Changes in NIV technology included a higher proportion of children using nasal masks, and minimal changes in both airway pressures and respiratory rate. While overall survival in this pediatric cohort was high at 95%, there was an increase in mortality rates for children using long-term NIV over time that may be attributable to the increase in the proportion of children with CNS and Cardio-Resp conditions starting long-term NIV. NIV discontinuation rates due to improvement of the underlying condition, decision to stop therapy, and transfer to adult services also increased over time.

The continued growth in the use of long-term NIV in children has been reported by groups around the world [5–10, 13]. Factors contributing to this increase likely include improvements in the available technology, progressive experience with this technology amongst healthcare providers, and increased use in children surviving critical conditions [14, 23, 24]. A high rate of survival, also seen in our cohort, is likely to contribute to on-going increases in the prevalence of this population and more resources needed to provide medical care, consistent with reports by other groups [6, 10, 25]. These trends are relevant not only to pediatric care but to the provision of adult healthcare services given the high likelihood of transition into adulthood.

Medical complexity and severity of sleep disordered breathing did not change over time in our cohort; this could be because children requiring long-term NIV are a priori a medically complex group. Definitions of children with medical complexity include children with significant chronic conditions affecting multiple body systems, progressive conditions associated with deteriorating health with limitations on life expectancy, continuous dependence on technology for at least 6 months, and malignancies that affect life function, with additional consideration of factors such as healthcare needs and usage, social and health system factors, and functional limitations [20, 26, 27]. In our cohort, almost all children had at least one additional comorbidity, one quarter were supported by one or more additional technology, and only 16% ceased NIV because of improvement, suggesting that a substantial portion of these children are medically complex. Children with medical complexity have higher healthcare utilization, longer hospital lengths of stay, higher attributable healthcare costs, and impact on families including sleep deprived parents/caregivers, and financial and social hardships [28–33]. Applying the frameworks of medical complexity to children using long-term NIV may be useful to differentiate both needs for care, relevant outcomes, and anticipated trajectories, including survival, for those with and without medical complexity.

While the overall rate of survival is high in our cohort, the increase in mortality across epochs is concerning. Despite there have not been significant changes in the overall level of complexity, severity of the sleep disordered breathing, and age at NIV initiation or death, mortality rate has grown. Interestingly, children that died while using NIV were on average younger than the median age for NIV initiation (3.4 vs 7.8 y), perhaps highlighting that younger children requiring NIV represent a distinct population. Further subgroup analysis will be required to determine specific predictors of mortality in this younger group which may include differences in underlying conditions or higher severity sleep disordered breathing. Our subgroup analysis confirmed increasing mortality trends for children with CNS conditions, which might be attributable to more children with these conditions starting on long-term NIV over time and lower survival. Children with Cardio-Resp conditions seem to show the same trend with an increasing number of children initiating NIV and lower survival curves. We did not demonstrate increasing medical complexity or clinically relevant changes in severity of sleep disordered breathing in both children with CNS or Cardio-Resp conditions. This may be because these have not changed or that our measures, including the fact that we did not categorize the severity of the underlying medical condition leading to NIV, were unable to capture changes in severity of medical illness. Multicenter prospective studies that allow larger sample size are indeed needed to confirm mortality rate and survival patterns. Two other cohorts reported on mortality rates by underlying medical condition. In one, after exclusion of children with neuromuscular diseases followed into adulthood, the highest mortality rate was in children with cardiac surgery, chronic lung disease, and ‘other’ conditions which include CNS conditions [6]. In the second, of 11 reported deaths, 4 (36%) were in children with lower airway or CNS conditions [9]. While there may be benefits of long-term NIV other than survival, counseling with respect to survival should differ by medical condition. Prospective tracking with systematic collection of outcomes beyond survival are needed to better define the benefits and risk for the use of long-term NIV in specific medical conditions in children.

In addition to an increase in mortality over time, NIV discontinuation for other reasons also rose; the explanation for these increases remains unclear. NIV discontinuation due to improvement of the underlying condition has been described previously, with higher likelihood to discontinue NIV in children with UA or chronic lung diseases compared to MSNM disorders [7–10, 12]. No prior reports described trend in NIV discontinuation because of family/patient decision to stop therapy for comparison with our results. Understanding the factors associated with improvements as well as decision to stop NIV will provide important information to children and their families for decision making at the time of initiation of long-term NIV.

There are some limitations of our study that must be acknowledged. Given the retrospective study design, data collection was limited to data available in the medical charts; if information on comorbidities or additional therapies was not documented, these may be underestimated in our cohort. Missing data, however, did not differ by diagnostic category or epoch so is unlikely to explain the reported trends. We defined long-term NIV as a minimum of three months use which excluded children who had difficulty initiating NIV. While we agree that the children who fail to initiate NIV are an important group to understand, a prospective study is needed to identify the characteristics of this group of children. Mortality rates reflect those children who expired while using NIV as those who discontinued for other reasons were not followed.

## Conclusions

This multicenter longitudinal study highlights the growing number of children receiving long-term NIV, consistent with trends worldwide. It demonstrates a greater use of long-term NIV in children with underlying CNS and Cardio-Resp conditions and small changes in technology use. Though overall survival remains high, an increase in mortality rate may be attributable to the shift in the underlying medical condition of children initiating long-term NIV and highlights differences in survival by diagnostic category. Children using long-term NIV likely have a high rate of medical complexity which has not changed over time. Additional understanding of the components of medical complexity, including healthcare usage and impact of families, will help to inform the care and resources needs as well as to set standards for the use of long-term NIV in children.
